# Communicating physical activity messages with adolescents: what works? A scoping review with stakeholder consultation

**DOI:** 10.1186/s12966-025-01717-8

**Published:** 2025-02-19

**Authors:** Caera L Grady, Elaine Murtagh, Kwok Ng, Enrique García Bengoechea, Catherine B Woods

**Affiliations:** 1 Physical Activity for Health Research Centre, Health Research Institute, University of Limerick, Limerick, Ireland; 2 Department of Physical Education and Sport Sciences, University of Limerick, Limerick, Ireland; 3 Faculty of Education, University of Turku, Rauma, Finland; 4 Institute of Innovation and Sports Science, Lithuanian Sports University, Kaunas, Lithuania; 5 Research & Innovation Unit, Sport Ireland, Dublin, Ireland

**Keywords:** Communication, Youth, Secondary school, Promotion, Consultation, Stakeholder

## Abstract

**Background:**

Global levels of adolescents’ physical inactivity are cause for concern, despite the well-documented health benefits of physical activity (PA). Addressing the messaging of PA is one approach to improve PA knowledge. While increased knowledge does not necessarily lead to behavior change, physically active students have better knowledge of the health benefits of PA. Recently, researchers have highlighted the need for an effective communication strategy for PA messages. This review aimed to summarize the state of the available evidence about the operationalization of communicating PA messages, the evaluation, and effectiveness of PA messages.

**Methods:**

The Levac six steps and Joanna Briggs Institute methodological guidance for scoping reviews were followed. Five databases were searched up until April 8th 2024. Both title and abstract and full-text screening were piloted whereby 10% of the total articles were double-screened and the remainder were completed by CG. Data were extracted and a data-based convergent synthesis design was used following qualitative synthesis methods. Finally, a consultation with key stakeholders was held to confirm the findings concerning practical relevance.

**Results:**

A total of 19,412 articles were identified from searches, 94 full texts were included in the final analysis, corresponding to 80 individual studies. The evidence confirms that there are many factors to consider when communicating PA messages and evaluating their effectiveness. Inconsistencies exist regarding the timing and frequency of message delivery and the evaluation of effective communication. When communicating PA with adolescents, messages commonly focus on the benefits of PA and strategies to overcome barriers and are commonly delivered in the school setting by researchers or school stakeholders i.e. teachers, peers. Messages should be concise, positively framed, support adolescent autonomy, and utilize different messaging platforms and techniques to avoid staleness.

**Conclusions:**

There is a lack of a standardized approach to communicating PA messages with adolescents making evaluation and comparison challenging. Future research should focus on developing guidance to facilitate the effective communication of PA messages with adolescents.

**Supplementary Information:**

The online version contains supplementary material available at 10.1186/s12966-025-01717-8.

## Background

Adolescents (10–19 years) account for 16% of the world population [1]. This period of rapid physical, social, emotional, and cognitive development is where PA behaviors and habits formed can be tracked into adulthood and impact individual health throughout the lifespan [2–4]. Despite the World Health Organization’s recommendation that adolescents should accumulate at least an average of 60-min per day of moderate to vigorous PA [5], to attain the well-documented health benefits, global trends of adolescents’ PA levels remain low [6].

Physical inactivity is a complex challenge and there are no single solutions. Growing evidence suggests the benefits of a whole-of-system approach to tackle PA behavior change [7, 8]. Despite being an ideal PA promotion setting, the school is a unique, complex, and adaptive sub-system that can cause difficulties when implementing such behavior change programs [8, 9]. Whole-of-school programs have been widely advocated for by researchers and international bodies such as the International Society of Physical Activity and Health and the World Health Organisation [7, 10, 11].

Physically active students are more likely to have better knowledge of PA and its’ benefits [12–15]. Knowledge of PA does not necessarily lead to improved behavior. However, receptance of the PA guidelines does influence attitudes, perceptions of capability, and intention to enact the guidelines [16]. Few adolescents between 11–18 years of age across Europe can identify the correct PA recommendations and there has been little change in this over time [12, 17–20].

The scale of adolescent physical inactivity combined with the low PA knowledge among adolescents may indicate issues with the methods used to promote or communicate PA messages with adolescents. A recent systematic review that explored stakeholders’ and end users’ perceptions of the PA and sedentary behavior guidelines highlighted that guidelines should include more lay language, definitions, and implementation strategies for communicating with end users [21]. Ireland was the first country to address this when updating their PA and sedentary behavior guidelines in 2024 and sought to develop PA messages for public and professional audiences [22].

PA messaging has been described as ‘the overall process of designing, creating, and delivering PA messages’ [23]. The research on PA messaging has been synthesized for the overall population, adults, parents, youth with disability, and underserved communities [23–28]. To the author’s knowledge, there has been no attempt to synthesize the PA messaging literature for adolescents thus far. In 2020 Milton et al. [29] suggested developing clear communication strategies to help the way PA messages are promoted and outlined a planning framework for PA communication. In response, a PA messaging framework and checklist were developed [30]. Both emphasized the importance of formative research before creating PA messages [29, 30].

This review addressed the following research questions: i) How is the communication of PA messages for adolescents operationalized? ii) What outcomes were measured to evaluate communicating PA messages with adolescents and what instruments were used? iii) What is the current state of evidence regarding the effectiveness of communicating PA messages with adolescents?

## Methods

This review was registered on the 18th March 2022 (OSF.IO/BCNS6) and a peer-reviewed protocol was published thereafter [31]. A rigorous approach was adopted following the Levac et al. [32] six steps, guidance from the Joanna Briggs Institute and the PRISMA-ScR checklist [33, 34] (Supplementary File 1).

### Search strategy

Inclusion criteria were devised after a preliminary search of the literature was conducted (Table 1) [31]. Five electronic databases were searched from 9th March 2022 to 8th April 2024: Scopus, EBSCOHost (CINAHL complete, Education Source), PubMed, and WHO Global Index Medicus (Table 1). Keywords and subject headings formed the search strategy which included three search strings: i) adolescents, ii) physical activity, and iii) messaging or communication (Supplementary file 2).

**Table 1:** Inclusion and exclusion criteria for screening

	**Include**
**Population**	Adolescents in second-level schools and, or between 10–19 years of age; adolescents with disabilities; parents of adolescents.
**Language**	Full text in English
**Year of publication**	1995- 2024
**Outcomes**	Studies that intend to change PA levels and discuss communicating PA with adolescents and, or discuss messaging, PA and adolescents.
**Source type**	Peer reviewed journal articles and grey literature (including theses/ dissertations, reports, conference abstracts and proceedings).
**Location**	Any

### Screening and selection

**Fig. 1:**
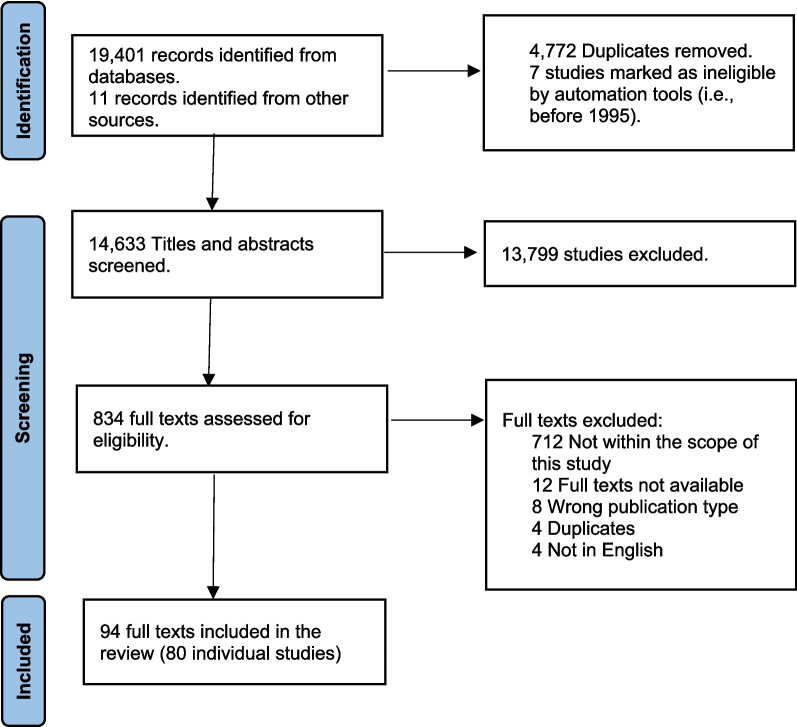
PRISMA flow diagram

Search results were downloaded to Endnote where duplicates were removed before uploading to Rayyan where further duplicates were identified and removed [31]. Before screening, automated tools were used to check that any publications before 1995 were removed. Both title and abstract and full-text screening followed the same process whereby 10% of articles were double screened (by CG, KN, EM, EGB) against eligibility criteria [31]. An agreement rate of 75% ended the screening process which was followed by a consensus meeting [31]. An independent reviewer (CW) acted as arbitrator for any discrepancies that remained and made the final decision. The PRISMA flow diagram displays the final numbers of studies that were included at each stage (Fig. 1).

### Data extraction

A data extraction sheet was developed including key study characteristics, methods, intervention details, underpinning theories, details of the communication of PA messages, and key findings. Two reviewers (CG and KMN) piloted data extraction independently with 10% of the included studies [31]. Discrepancies were discussed and amendments agreed before one author (CG) proceeded with the remaining data extraction. The characteristics of the included studies were summarized and presented to the review team (CW, EM) to reach agreement before proceeding with data synthesis.

### Data synthesis

Consistent with the aims of this study, its’ scoping nature, and the approach commonly used in mixed studies reviews with diverse designs, a narrative synthesis approach was deemed appropriate [35]. A data-based convergent synthesis design was used following qualitative synthesis methods [35]. Data were narratively synthesized as per the elements in the framework outlined by Popay et al. [36]. A preliminary synthesis was completed to organize results and identify patterns [36]. Data were read and re-read by the first author to ensure familiarization. Data were grouped and named to represent the data. Next, the first author looked for factors to explain the differences and similarities within and between studies to understand the effect of a particular intervention [36]. Neither theory development nor assessing the robustness of the synthesis were carried out due to the exploratory nature of this review [36]. To understand the current state of the evidence regarding the effectiveness of communicating PA messages, experimental studies were examined (i.e. randomized control trial (RCT), quasi-experimental, or pilot or feasibility), and results were reported as a change or no change on the various outcome measures. Findings were presented under each research question. Finally, a summary table of future recommendations for research, practice, and policy was developed based on recommendations or good practices from the included studies.

### Stakeholder consultation

As per the final step in the scoping review framework, a stakeholder consultation was held to confirm the findings of the review [32]. Ethical approval (University of Limerick Education and Health Sciences Research Ethics Committee EHS_2023_04_08_EHS) and written informed consent was obtained before commencing. A focus group discussion facilitated by CG, was held at a local secondary school with an online link for the international expert. A purposeful sample of six stakeholders involved in the promotion of PA to adolescents were recruited (international researcher (*n* = 1), policy maker (*n* = 1), practitioner (*n* = 1), school principal (*n* = 1), and adolescents (*n* = 2)) [32]. Following a presentation of the scoping review methods and key findings, the stakeholders could comment on or ask questions about the review. A semi-structured interview script guided the discussion where stakeholders were asked to identify any i) findings that stood out to them, ii) similarities, and iii) differences between the findings and their experience with communicating PA messages with adolescents.

The discussion was recorded and an assistant moderator (KMN) took notes. Stakeholders had an opportunity to ask questions during the focus group, review a summary of the discussion, and add any further comments up until two weeks later. Focus group reporting followed the Consolidated criteria for Reporting Qualitative research (COREQ) checklist (see availability of data and materials). Full reporting of focus group procedures is available in the Open Science Framework online repository (see availability of data and materials).

The first author listened back to the recording, transcribed, and summarized the discussion (accuracy confirmed KMN) before sharing with participants. Data were narratively synthesized and mapped to the review questions.

## Results

### Description of studies

As outlined in Fig. 1, 19,412 sources were identified from searches, 4,772 were duplicates, and seven were identified as ineligible by automated tools within Rayyan prior to screening. After title and abstract screening 834 proceeded for full text review which resulted in 94 sources being included for analysis.

### Characteristics of the included studies

Overall, 94 publications representing 80 different studies were included in this review. Of the 94 publications, 91 were journal articles and the remaining included a conference proceedings paper, a brief report, and a thesis. Seventy percent were published in the last 10 years. Eight of the 80 studies had multiple publications; Trial of Activity in Adolescent Girls (TAAG) [37–40]; Girls on the Move program [41–43]; the HEALTHY study [44–48]; an SMS-based intervention promoting PA to adolescents in Hong Kong [49, 50]; the Adolescent Teen Leader Avoiding Screen-time (ATLAS) study [51, 52], the effect of activity trackers and text messaging on exercise, fitness, and PA on self-efficacy study [53, 54], Fit24 [55, 56] and the “Som la Pera” study [57, 58].

Table 2 outlines the study location and participant demographics. The USA accounted for almost 40% of studies whereas 10%, 7%, and 5% were from the UK, Australia, and Spain respectively (*N* = 80). The countries listed as ‘other’ were those that had less than or equal to five studies.

**Table 2:** Characteristics of included studies and demographics of participants (*N* = 80)

Characteristics	n	% (of total *N* = 80)
**Study location**		
USA	31	39.0
UK	10	12.7
Australia	7	8.9
Spain	5	6.3
Other	26	32.9
**Participant demographics**		
**Gender**		
Females only	10	12.7
Males only	2	2.5
Mixed genders	67	84.8
**Other populations/groups**		
Physical disability	2	2.5
Intellectual disability e.g. ADHD	1	1.3
At risk of morbidity i.e. obesity, diabetes, CHD	6	7.6
Low-income/socio-economic community	2	2.5
Low activity level	3	3.8
Parents of adolescents	10	12.7
Other key adult informants (not specified)	1	1.3
**Race or ethnicity reported**^a^		
Mixed/ range of ethnicities	6	7.6
Asian or pacific islander	4	5.0
Latin America/ Hispanic	11	13.9

^a^Lau et al. [50] (SMS-based intervention for PA promotion), Lubans et al. [52] (ATLAS), Schneider et al. [48] (HEALTHY), and Soltero et al. [55] (Fit24) were part of larger studies that had both experimental and non-experimental outputs

^b^Counted if specified in title or participant section of manuscript

**Fig. 2:**
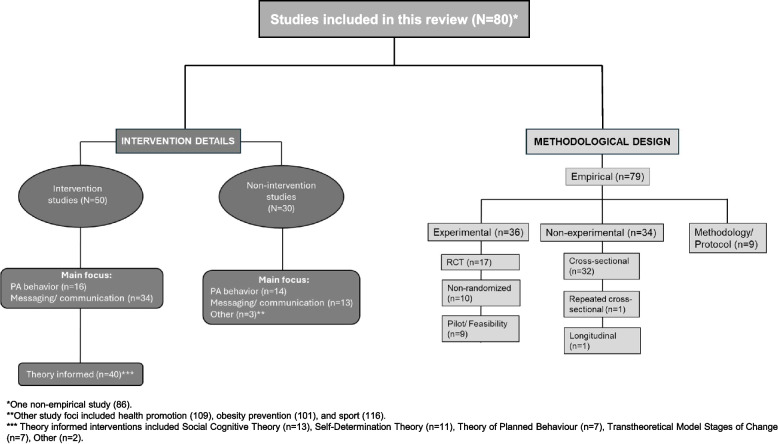
Methodological design and intervention details of the included studies

The methodological design and intervention details of the included studies are outlined in Fig. 2. Of the 80 studies, 79 were empirical research and the one non-empirical research was a case study reporting on three school-based health promotion efforts in Denmark [59]. The empirical research studies represented 34 non-experimental or observational research designs, 36 experimental research designs, and nine methodological or protocol studies. Three of the eight studies with multiple publications had both experimental and non-experimental outputs, with three conducting a RCT (TAAG, HEALTHY, and ATLAS) and the remaining seven publishing methodological or protocol studies.

Fifty of the 80 studies involved an intervention, of which the majority were messaging or communications interventions (62.5%). Eighty percent of studies that had an intervention were underpinned by a theory, model, or framework and the most commonly used are outlined in Fig. 2.

### Narrative synthesis of findings

#### Operationalization of communicating PA messages

To understand the operationalization of communicating PA messages, data were summarized from all eighty studies in relation to the content, context, and mode in which PA messages were delivered.

##### Message content

Most studies (*n* = 23) outlined the benefits of regular PA (physical and mental health, enjoyment, social, etc.) [39, 60–63], 10 addressed barriers to engaging in PA [55, 57, 58, 63–70], and seven provided suggestions or tips for PA e.g. how much and what intensity [52, 55, 69, 71–74]. Eleven studies provided educational or skill-building information [20, 59, 60, 75–82], eight provided feedback on participants PA levels [41, 55, 58, 68, 73, 78, 81, 83], and motivational messages were used in six studies [41, 51, 57, 58, 75, 82]. Finally, making friends and engaging in PA with family members, professionals, and peers were mentioned in two studies [83, 84].

Message banks were used in six studies. Message banks were typically developed using psychosocial theories and models such as the Transtheoretical Model (TTM) and Social Cognitive Theory or were co-created with adolescents [50, 55, 60–85]. For example, the Chicas Fuertes study had a bank of 330 messages addressing different levels of psychosocial and environmental factors affecting PA such as the TTM stages of change, decisional balance, and self-efficacy [61]. Messages were then individually tailored based on whether scores on these variables increased, decreased, or remained stagnant [61]. Two studies used co-creation or qualitative methods with adolescents to develop a PA message bank [50, 85]. One example by Raeside and colleagues utilized both end-user input and relevant guidelines and behavior change theories [86].

##### Context in which the message was delivered (Where and When)

The school was the most common setting in which messages were delivered (*n* = 31) [17, 38, 41, 48, 49, 52, 59, 60, 62, 64, 66, 73, 75, 76, 78, 83, 87–100], followed by the home (*n* = 11) [56, 65, 79, 80, 85, 101–106], community (*n* = 9) [68, 82, 107–113], and those delivered in multiple settings (*n* = 2) [114, 115]. A setting was not specified or not applicable in 26 studies e.g. online or text message intervention. Many of the above (*n* = 37) also had a digital component where messages were delivered through social media, SMS, emails, etc. Messages were mainly communicated during school hours (*n* = 19) [17, 38, 43, 48, 57–88, 90–96, 99], some were before or after school (*n* = 13) [49, 50, 56, 60, 62, 71, 74, 79, 82, 86, 97, 112, 116], and one was both during and outside school (*n* = 1) [41]. Ten did not specify a time [65, 68, 72, 102, 103, 107, 109, 111, 113, 115]. Frequency of message delivery ranged from once to multiple times per week (*n* = 6) [80, 85, 86, 104, 108, 112], once to multiple times per day (*n* = 6) [55, 56, 68, 79, 82, 101], or during daily life at home (*n* = 2) [105, 106]. Two studies stated that timing and frequency should support adolescent autonomy [50, 84].

##### Mode of delivery (Who)

Mostly researchers were the key communicators whether directly or indirectly (online) (*n* = 30) [54, 55, 60–62, 66, 67, 69, 71, 74, 76, 77, 79, 82–87, 99–120]. Twelve studies had a variety of stakeholders as communicators [48, 51, 64, 68, 69, 78, 81, 89, 93, 107, 111, 114] and eight used teachers or service providers (school or community) [17, 38, 50, 59, 75, 88, 95, 113]. A further eight used peer-to-peer communication [72, 73, 80, 90, 91, 94, 97, 121], three used parents or family members [105, 106, 122], and seven included other communicators e.g. healthcare practitioners, the social or cultural environment, or public health campaigns [41, 65, 80, 96, 98, 109, 110].

##### Mode of delivery (How)

Studies that addressed the tone of delivery agreed that it should be positive, empowering, autonomously supportive, upbeat, humorous, and if delivered by adults, friendly but professional [20, 69, 71, 81, 106, 117] e.g. use emojis, GIFs, and exclamation points [82]. Emphasis should be placed on having fun, the social aspect of PA, and ensuring it is not too competitive [20]. It was also highlighted that it is important to avoid negativity and nagging [114] and to be inclusive and diverse [69, 107, 108, 113]. For the style of delivery, two studies indicated that messages should be age-appropriate and visuals should be colorful, bright, and attractive [61, 63, 69]. App interfaces need to have a user-friendly experience and the design of PA technology is important [63]. In addition, images should be relatable, inclusive, and diverse for body shape, ethnicity, and racial representations [37, 107]. The format of the messages should be concise, not too complex, and should be delivered through a variety of mechanisms or platforms, including print (posters, flyers, etc.), oral (announcements, workshops, etc.), digital (Television, electronic billboard, etc.), or online (social media, websites, etc.) [46, 63, 69, 79, 87].

##### Mode of delivery (Methods used)

Studies outlined many ways to grab the attention of the message receiver. Individualizing messages was observed in seven studies e.g. message tailoring [61, 75, 76, 79, 88, 117, 123]. Utilizing the existing environment was also referred to in six studies [44, 48, 64, 95, 96, 103], and understanding the target audiences’ existing levels of knowledge when communicating PA messages was emphasized in four studies [77, 100, 109, 111]. Eight studies used tools and skills to help improve the communication of PA messages with adolescents [49, 56, 57, 61, 66, 89, 100, 106] while six focused on an educational component to increase knowledge and understanding with the aim of improving behavior [44, 59, 66, 78, 91, 116].

#### Outcomes measured and instruments used to evaluate the communication of PA messages

Of the 80 studies in this review, 48 utilized various outcomes and instruments to evaluate the communication of PA messages. Four main categories of outcome measures were identified including i. PA behavior change, ii. PA knowledge, awareness, and attitudes, iii. psychosocial functioning, and iv. intervention engagement and exposure rates. Furthermore, various instruments were used to evaluate each outcome measure. Twenty-one different types of self-report PA questionnaires and 10 different types of device-based measures were used to measure PA behavior change. Eight different instruments were used to assess PA knowledge, three for awareness of PA, and four for attitudes towards PA. With regards to psychosocial functioning, nine instruments used to assess social support, seven for PA self-efficacy, four for PA intentions, four for subjective norms, four for PA motivation, two for perceived behavioral control, and one for PA planning. For intervention engagement, there were 11 instruments and five instruments were used to assess intervention exposure. Supplementary file 3 outlines the various outcome measures and instruments used across the included studies.

#### Current state of the evidence around the effectiveness of PA communication interventions

Of the 50 intervention studies reviewed, 36 were eligible to be included in this section of the synthesis i.e. were an experimental design. Overall we found various outcome measures, as listed in the above section, that were used to evaluate the effectiveness of communicating PA messages. Each measure was examined for evidence of effect (i.e. a change or no change) that the PA communication intervention had on the outcome measure. Due to the array of instruments used across the studies and varying study designs, the following results should be interpreted with caution. A supplementary table is provided which summarizses the information from the 17 included RCT to allow further interpretation of the types of studies and outcomes (supplementary file 4).

##### Self-reported PA behavior change

No change in PA levels were reported in a range of studies that measured PA using self-report instruments. The studies that did not report a change in self-reported PA included those that i. used persuasive communication [75, 99, 117], ii. had an internet PA program tailored to adolescents’ stage of change, and daily text messages [50], iii. had a school-based educational intervention [89], iv. had a social marketing or communications campaign [48, 93], v. involved providing health-promoting information digitally (e.g. website, tv, sms) or monthly social support group meetings [80, 119, 124], and vi. sent motivational text messages to reach a step goal [125].

Studies that did report a positive change in self-reported PA mainly all included an element of message tailoring or framing. Such as those that used i. messages emphasizing affective gains of PA for low-active adolescents [62], ii. a gamification-based goal targetting intervention at school [104], iii. a WhatsApp group tailored to TTM stages of change [67], iii. an educational intervention embedded into the curriculum with targeted information delivery and structured PA sessions [78], and iv. Socio-ecological model targetted environmental activities and educational lessons [96]. The others included behaviorally focused computer-based education as opposed to traditional education methods [126], and a social marketing intervention with PA challenges and a campaign information exhibition to engage adolescents [58].

##### Device-based measurement of PA behavior change

No changes in adolescents’ PA levels were reported in most studies that used a device-based measure of PA (i.e. accelerometer, activity tracker, or pedometer). Including studies that i. had a communications element as part of a multi-component school-based intervention [43, 52, 92, 100] (accelerometer-measured PA), ii. used text messages and activity trackers to self-monitor PA levels [54, 112] (PA measured with activity tracker and accelerometer respectively), iii. used technology probes, nudges, or alarms to remind adolescents to be active [82, 103, 116] (one study measured PA with an activity tracker and two with pedometers respectively), and v. used goal-framed messages and PA planning [83] (accelerometer-measured PA).

The studies that did report a change in device-based measures of PA all included an element of self-monitoring activity levels and all used different types of devices. These included i. tailored messages based on adolescents’ goals and self-reported activity levels (accelerometer-measured PA) [84], ii. adolescents self-monitoring their PA, an online educational program and bi-weekly text messages (PA measured with activity tracker) [108], and iii. a mHealth linked wearable activity tracker and a facebook group for social interaction (PA measured with a pedometer) [76].

##### Knowledge, awareness, and attitudes towards PA

Knowledge of PA improved in all studies that it was measured in, all of which had an educational intervention component [78, 89, 109, 126]. More specifically these studies involved i. videos on the importance of being physically active and eating a balanced diet [89, 109], ii. a combination of educational materials (e.g. presentations, videos), an activity book, parental information sessions, and structured PA sessions [78], and iii. computer-based educational information [126].

Awareness was mainly assessed as awareness of the intervention that shared the PA message however, there was no comparison group to establish whether the intervention had an effect [48, 96].

Attitudes towards PA improved in studies that used i. persuasive communication [75, 117], ii. behaviorally focused education [78], and iii. goal framing (extrinsic and intrinsic conditions showed no difference) [83]. Attitudes towards meeting the PA guidelines did not change based on branding of the guidelines [118].

##### Self-efficacy for PA

Most of the studies did not see a change in PA self-efficacy including those that had i. a multi-component school and/or home-based intervention [43, 100], ii. digital health nudging, and iii. used text messages and activity trackers to self-monitor PA levels [54]. Those that did show an increase in PA self-efficacy included a WhatsApp group tailored to TTM stages of change [67] and a technology-based intervention [108].

##### PA motivation

Changes in PA motivation were observed in studies that had a school and home-based intervention [100] and a smartphone application to promote PA and reduce screentime [52].

##### Social support for PA

Social support for PA increased in a study that compared computer and traditional educational methods [126] but did not change in a study that used a school and home-based intervention [100].

##### Subjective norms for PA

Most of the studies that measured subjective norms did not change adolescents’ normative beliefs. This included studies that used persuasive communication and planning [117] and messages targetting salient and non-salient behavioral beliefs [99]. Whereas a study that used theory-based messages with cognitive prompts in the classroom did improve adolescents’ subjective norms for PA [75].

##### PA intentions

Changes in adolescents’ intentions towards PA were reported in a range of studies including those that used persuasive communication [75, 117] and message framing [52]. No change in adolescents’ intentions towards achieving the PA guidelines were observed based on the branding of the guidelines [118].

##### Intervention engagement and exposure rates

Intervention engagement and exposure rates were generally discussed together in studies but few studies measured engagement and exposure in a comparision group. The studies that did not compare to a control group included those that i. delivered messages directly to the adolescents (e.g. SMS or classroom health discussions) [50, 66], ii. involved both parent and adolescent participation [100], or iii. involved an online, app, or SMS-based interventions [49, 51] all of which had lower engagement rates than intended. A school-based intervention reported greater exposure when delivered by University researchers as opposed to the school-based program champions [40]. The studies that did have a comparison group found that there were no differences in engagement between the groups this included interventions that used an internet PA program and generic text messages [60] and used television advertisements to share health-promoting messages [124].

To summarize the current state of the evidence regarding the effectiveness of PA communication interventions, comparison between the studies measures and methods are difficult due to the diverse methods and instruments used (supplementary file 3). However in general we found, irrespective of the type of intervention there were little changes on adolescents’ PA behavior, some increases in their knowledge, awareness, and attitudes towards PA, and positive changes on PA self-efficacy, motivations, and intentions for PA, and little changes in subjective norms.

#### Consultation with key stakeholders

The stakeholders reflected on the similarities and differences to their own practices particularly in relation to the operationalization of communicating PA messages. Similar to the evidence presented, in practice it is important to portray *“a clear message”, “straightforward”* not “*too complicated”*, to engage with adolescents, to understand the perceived barriers to PA, and to bring a “*positive energy*” (Male secondary school senior student). Among the practitioners (*n* = 2) they confirmed that despite having these *“lovely posters or these posts on social media, different ways of promoting” it is still “not influencing the behavior, it did not change” which leaves unanswered questions such as* “*how could we do it differently, like or what could we or how else we could promote it*?” (Secondary school Physical Education Teacher, program implementor, female)*.*

Stakeholders suggested that PA messages should target changing attitudes towards PA away from those feelings that *“you have to be good at it, that you have to play already”* which often results in non-participation (Female secondary school senior student) or the stigma associated with *“being sporty and being involved defined you in a way… very non feminine”* (Female policymaker) is one such approach. Additionally, overcoming *“the sports driven model [that] has been in schools for a long time”* (Female policymaker) which can prevent adolescents from engaging with PA messages and the behavior*.* Another similarity included the need to consider the target group, how “*they hear the message, and how they understand, is it relevant to them?”* (Female policymaker).

One key factor that was missing was the impact of indirect messages, *“the message we give out as coaches or teachers”* that may lead to people not engaging in *“sport and physical activity due to the impact of the negative experience at the coaching level”* and this comes down to the “*poor behavior as a coach and those messages have a huge impact”* (Male secondary school senior management)*.*

In relation to evaluating the communication of PA messages, the stakeholders were shocked by the studies that showed a lack of change on PA behavior. Improving adolescents’ knowledge and awareness of PA through communicating PA messages was noted as a potentially worthwhile investment. The “*complexity of obviously what you’re getting into”* with PA behavior change was recognized by the stakeholders and how *“it is a much longer-term investment piece”* and how it may be worthwhile to “*focus on the easy wins and the things that are obvious [knowledge, acceptability, exposure to messages] that that will ultimately support the bigger piece ticket items like behavioral change”* (Female policymaker)*.* Further supporting quotes can be found in Supplementary file 5.

### Recommendations for communicating PA messages with adolescents

To summarize the findings presented above and inform actionable recommendations for research, practice, and policy Table 3 outlines some key recommendations to consider when communicating PA messages with adolescents.

**Table 3:** Recommendations for communicating PA messages with adolescents

Responsibility	Recommendation
**Research**	Develop a tool or mechanism to standardize the evaluation of communicating PA messages effectively e.g. a standardized evaluation framework.
	Examine the long-term impact of communicating PA messages on PA knowledge, awareness, and attitudes.
	Explore the role of indirect messages and their impact on adolescents PA.
	Determine a suitable and “effective” time and frequency in which PA messages are delivered.
	Consider scale-up and sustainability of intervention delivery from the beginning e.g. less researcher dependent, more end-user communicators such as peers, social influences, or role models such as coaches, teachers, etc.
	When sufficient evidence exists, systematically review and meta-analyse the evidence of each type of intervention that communicates PA messages in relation to their effectiveness e.g. SMS-based interventions, digital technology interventions, and communication or social marketing campaigns.
**Practice**	Embed best practices for communicating PA messages within existing multi-component school-based programs to maximise the impact of the program.
	Consider inclusivity and diversity when delivering PA messages
	Consider the adolescents’ autonomy during decision-making.
	PA message content: Use tailored and gain-framed messages that focus on the benefits of PA and provide strategies and tips to overcome the barriers of PA were deemed most promising.
	Mode of delivery: Messages should be positive, empowering and age appropriate and should use various platforms such as print, oral, digital, and online.
**Policy**	Advocate for the use of a standardized approach to evaluating efforts for communicating PA messages
	Strengthen research and evaluation capacity to inform effective policy solutions
	Develop, or advocate for, a PA message communication framework or plan to streamline the methods, or techniques used to communicate PA messages to facilitate comparisons between strategies and mechanisms used.

## Discussion

### Statement of principal findings

This review exposed a great degree of disparity between studies about when and how PA messages should be communicated with adolescents. However, there was a high level of agreement concerning who, what, and where PA messages should be communicated. Furthermore, inconsistencies were found with evaluating PA messages thus, determining the extent of the impact, the strategies, and approaches used within studies had on adolescents was challenging. Nevertheless, this review provides key learnings for researchers, practitioners, and policymakers alike regarding the communication of PA messages to date and the future of PA messaging with adolescents. Due to the distinct physical, social, emotional, and cognitive changes that take place during this life stage, this study focused solely on adolescents aged 10–19 years to build on previous PA messaging reviews [4, 23, 25, 27].

### Comparison to related research

The content, context, and mode in which PA messages are delivered are essential to understand the operationalization of communicating PA messages. Firstly, regarding the content of the messages the findings in this review were similar to those reported in other reviews [23, 25–28]. For example, including information about the benefits of PA when communicating PA messages with different populations was important [23, 25–28]. Other commonly cited PA message content included barriers to PA, suggestions for PA, feedback on PA levels, and other educational information. These topics appear similar across all population groups. Adolescents also require content that is engaging, age-appropriate, and considers their needs and desires such as, fun and enjoyment [23, 27]. The stakeholder consultation highlighted the lack of evidence and guidance around indirect messages communicated to adolescents which highlights an area for further investigation.

The school setting was the most common place where and when messages were communicated; however, the home and community were also prominent. This finding is not surprising considering the school is considered an ideal PA promotion setting for adolescents [8, 11]. Common times to deliver messages were either during or outside of school hours. Timing and frequency were largely inconsistent across the studies included in this review. Factors such as the timing of receiving the PA message rarely considered adolescents’ autonomy. Autonomy is one of three basic psychological needs for motivation and personal growth and should be considered when communicating PA messages with adolescents [127].

The mode of delivery included who the communicator of the message was, how it was communicated, and the methods used to communicate. Researchers’ involvement in communicating PA messages, either directly or indirectly, occurred more frequently than any other stakeholders. It may be worthwhile to consider adolescents’ autonomy in relation to the delivery of PA messages, they may prefer peers or role models. For example, McHale et al. [128] concluded that younger adolescents can be effectively led by both older and same-age peers. Furthermore, a best practice statement highlighted that adolescents should be central to the communication process [129]. This differs from findings related to children and young people in another PA messaging review which suggested that adults were preferred which may indicate the need to consider children and adolescents separately when communicating PA messages [23].

When delivering a PA message, the tone, style, mechanisms, and platform are all important aspects to be considered. The tone being positively or gain-framed and empowering and the style being concise was a finding that complies with existing PA messaging reviews [23, 25, 26]. For example, Wright et al. [98] stated that young males and females interpret information differently and this should be considered during communication efforts. It should be noted that few studies broached the topic of diversity and inclusivity when communicating messages including culture, religion, ethnicity, gender, or the differing abilities of adolescents and this topic was important to those from minority backgrounds or with low activity levels [40, 69, 115].

The methods used to communicate PA messages were similar to that of previous reviews. Message framing, tailoring, and targeting were also referred to by Latimer et al. [26] and Williamson et al. [23] who both found that gain-framed messages that can be tailored or targeted to a specific audience are favorable. The consequences of studies using varying methods or techniques have not yet been addressed within the literature which presents a challenge for evaluation and comparison between studies and methods. Measures examining behavior change, knowledge, awareness, attitudes, and psychosocial functioning to evaluate the communication of PA messages were reported. Latimer-Cheung et al. [26] reviewed approaches for constructing PA messages to change self-efficacy and the findings showed promise of improved PA self-efficacy. Nevertheless, the array of measures found poses a challenge for comparison thus, there is a need to standardize the evaluation of communicating PA messages.

To understand the current state of the evidence on the effectiveness of communicating PA messages with adolescents, the various methods and approaches used in the studies were mapped to the outcomes measured to help identify any changes. This process revealed similar findings to the available literature. For example, a recent umbrella review of PA promoting mass media campaigns found that they were effective at increasing PA awareness and knowledge but have little impact on behavior change without community engagement or making environmental changes [130]. This aligns with this study’s findings of positive changes in knowledge, awareness, and attitudes but little changes on PA behavior. Furthermore, the consultation with secondary school stakeholders in this paper confirmed that changing PA behavior is a complex challenge and cannot be fixed by communicating PA messages alone. For example, Patrick et al. [80] compared three modalities of delivering information to promote weight loss in adolescents, to usual care in the USA, all had decreases in sedentary behavior but were not sufficient to increase PA. Primary outcome measures focusing on knowledge and awareness may be more appropriate rather than expecting PA behavior change from communicating PA messages alone. Garcia et al. [131] showed that if adolescents had more knowledge of a healthy lifestyle (not just PA), they were more likely to engage in at least one hour of PA a week. However, the long-term effects of improving knowledge, awareness, beliefs, and attitudes towards PA on changing behavior are not clear.

Despite observing some positive increases in measures of self-efficacy and PA intentions there is currently not enough evidence to determine if psychosocial functioning measures are impacted by communicating PA messages. Furthermore, during the consultation, stakeholders outlined the lack of attention towards adolescents’ affective responses to the messages they receive. Nevertheless, it may be worthwhile embedding best practices for communicating PA messages within existing multi-component interventions.

### Strengths and limitations

This scoping review provides the first attempt at examining solely the adolescent population in relation to considerations for communicating PA messages. Strengths include the systematic and transparent methods used starting with pre-registration (OSF.IO/BCNS6) and publishing a peer-reviewed protocol [31]. A rigorous search process was followed using broad inclusion criteria, five electronic databases, and checking reference lists of other reviews. All six steps of the scoping review framework were followed in the conduct of this review which is noteworthy as the sixth step of ‘consulting with key stakeholders’ is often neglected in scoping reviews [32, 132]. Finally, this review provides recommendations to advance research, practice, and policy for communicating PA messages.

We did not set out to appraise the quality of evidence included [31]. Some studies that had a broader age category, but would have been otherwise relevant, were excluded e.g. the VERB! It’s what you do [133] and WIXX [134] campaigns. The grey literature search was limited to databases that index grey literature therefore, some other relevant sources may have been missed. Similarly, study selection was limited to those published in English only. Overall, comparison between interventions in relation to their impact was limited due to the varying instruments and methodologies used. Finally, the consultation with key stakeholders was limited to a single focus group.

## Conclusions

This review outlined the PA messaging research to date, the limitations, and existing gaps for the adolescent population. The lack of a standardized approach to i) communicating PA messages with adolescents and ii) evaluating the communication of these messages makes comparison between studies challenging. Guidance is needed to facilitate the communication of PA messages with adolescents which could facilitate existing PA promotion efforts by policy makers and practitioners. Furthermore, there is a need to develop a measure or battery of instruments for evaluating the effect of the PA message that is communicated. Finally, future research should incorporate the ‘adolescent voice’ and autonomy when developing PA messages to ensure they are meeting the target population needs and desires.

## Supplementary Information

Supplementary Material 1.

## Data Availability

Data and materials are available in the Open Science Framework online repository including: - Focus group procedures - COREQ checklist - Results of individual sources relevant to research questions (Link to be included upon acceptance of publication) https://osf.io/mpq8u/?view_only=89dbc0ecf77742e38568b7423aba3fe5.

## Abbreviations

ATLAS: Adolescent Teen Leader Avoiding Screen-time
COREQ: Consolidated criteria for Reporting Qualitative Research
GOTM: Girls on the Move
PA: Physical Activity
PRISMA-ScR: Preferred Reporting Items for Systematic Reviews and Meta-Analyses extension for Scoping Reviews
RCT: Randomized Control Trial
TTM: Transtheoretical Model
TAAG: Trial of Activity in Adolescent Girls
WHO: World Health Organization
