# Supplementary material for: Communicating physical activity messages with adolescents: what works? A scoping review with stakeholder consultation
Source: Int J Behav Nutr Phys Act. 2025 Feb 19;22:20. doi: 10.1186/s12966-025-01717-8 (PMC11841338; doi:10.1186/s12966-025-01717-8)
Supplement: Supplementary file 1 — Supplementary Material 1. [file 12966_2025_1717_MOESM1_ESM.docx]

**Additional Files.**

*Supplementary File 1- PRISMA-ScR checklist*

| **SECTION** | **ITEM** | **PRISMA-ScR CHECKLIST ITEM** | **REPORTED ON PAGE #** |
| --- | --- | --- | --- |
| **TITLE** | | | |
| Title | 1 | Identify the report as a scoping review. | 1 |
| **ABSTRACT** | | | |
| Structured summary | 2 | Provide a structured summary that includes (as applicable): background, objectives, eligibility criteria, sources of evidence, charting methods, results, and conclusions that relate to the review questions and objectives. | 1 |
| **INTRODUCTION** | | | |
| Rationale | 3 | Describe the rationale for the review in the context of what is already known. Explain why the review questions/objectives lend themselves to a scoping review approach. | 2-3 |
| Objectives | 4 | Provide an explicit statement of the questions and objectives being addressed with reference to their key elements (e.g., population or participants, concepts, and context) or other relevant key elements used to conceptualize the review questions and/or objectives. | 2 |
| **METHODS** | | | |
| Protocol and registration | 5 | Indicate whether a review protocol exists; state if and where it can be accessed (e.g., a Web address); and if available, provide registration information, including the registration number. | 2 |
| Eligibility criteria | 6 | Specify characteristics of the sources of evidence used as eligibility criteria (e.g., years considered, language, and publication status), and provide a rationale. | 2 (Table 1) |
| Information sources* | 7 | Describe all information sources in the search (e.g., databases with dates of coverage and contact with authors to identify additional sources), as well as the date the most recent search was executed. | 2 |
| Search | 8 | Present the full electronic search strategy for at least 1 database, including any limits used, such that it could be repeated. | Supplementary file 2 |
| Selection of sources of evidence† | 9 | State the process for selecting sources of evidence (i.e., screening and eligibility) included in the scoping review. | 3 |
| Data charting process‡ | 10 | Describe the methods of charting data from the included sources of evidence (e.g., calibrated forms or forms that have been tested by the team before their use, and whether data charting was done independently or in duplicate) and any processes for obtaining and confirming data from investigators. | 63 |
| Data items | 11 | List and define all variables for which data were sought and any assumptions and simplifications made. | 3 & Supplementary file 4 |
| Critical appraisal of individual sources of evidence§ | 12 | If done, provide a rationale for conducting a critical appraisal of included sources of evidence; describe the methods used and how this information was used in any data synthesis (if appropriate). | N/A |
| Synthesis of results | 13 | Describe the methods of handling and summarizing the data that were charted. | 3-4 |
| **RESULTS** | | | |
| Selection of sources of evidence | 14 | Give numbers of sources of evidence screened, assessed for eligibility, and included in the review, with reasons for exclusions at each stage, ideally using a flow diagram. | 3 (Figure 1) |
| Characteristics of sources of evidence | 15 | For each source of evidence, present characteristics for which data were charted and provide the citations. | 4-5 (Table 2) |
| Critical appraisal within sources of evidence | 16 | If done, present data on critical appraisal of included sources of evidence (see item 12). | N/A |
| Results of individual sources of evidence | 17 | For each included source of evidence, present the relevant data that were charted that relate to the review questions and objectives. | Supplementary file 4 & Availability of data and materials |
| Synthesis of results | 18 | Summarize and/or present the charting results as they relate to the review questions and objectives. | 5-9 |
| **DISCUSSION** | | | |
| Summary of evidence | 19 | Summarize the main results (including an overview of concepts, themes, and types of evidence available), link to the review questions and objectives, and consider the relevance to key groups. | 9-11 |
| Limitations | 20 | Discuss the limitations of the scoping review process. | 11 |
| Conclusions | 21 | Provide a general interpretation of the results with respect to the review questions and objectives, as well as potential implications and/or next steps. | 11 |
| **FUNDING** | | | |
| Funding | 22 | Describe sources of funding for the included sources of evidence, as well as sources of funding for the scoping review. Describe the role of the funders of the scoping review. | 11 |

*Supplementary File 2- Full search strategy for CINAHL database (29th March 2022)*

| Search # | Search terms |
| --- | --- |
| 1 | (MM "Physical Activity") OR (MM "Exercise") OR (MM "Physical Fitness") OR (MM "Sports") |
| 2 | TI ( physical activity or exercise or physical fitness or sport ) OR AB ( physical activity or exercise or physical fitness or sport ) |
| 3 | S1 OR S2 |
| 4 | TX adolescents or adolescence or teenagers or teen or young people or youth or young adults |
| 5 | TX communicat* or messag* |
| 6 | S3 AND S4 AND S5 |

*Supplementary file 3- Outcome measures and instruments used throughout included studies*

| **Outcome measure** | **Method of measurement** | **Instruments (studies used, N=57)** |
| --- | --- | --- |
| PA behavior change | Self-report | International PA Questionnaire (72, 77) |
|  |  | International PA Questionnaire for Adolescents (63, 79, 106) |
|  |  | PA Questionnaire for Older Children (38, 49, 59) |
|  |  | PA Questionnaire for Children (50) |
|  |  | Researcher developed:   - Days and minutes spent exercising (64) - Hours physically active per day (123) |
|  |  | At school physical activity (109) |
|  |  | Practice of PA in leisure time (40, 82, 108) |
|  |  | Modified National Board of Health and Welfare in Sweden PA questionnaire (67) |
|  |  | Single item- PA measure (20, 73, 74, 76, 124, 125) |
|  |  | Single-item exercise measure (71) |
|  |  | Leisure time exercise questionnaire (69) |
|  |  | Self-administered PA checklist (46) |
|  |  | 5-items from CDC Youth Risk Behavior Surveillance System survey (120) |
|  |  | 11-items from National Health Interview Survey-Youth Risk Behavior Survey (100) |
|  |  | 2-items from School Physical Activity and Nutrition Questionnaire (103) |
|  |  | PA questionnaire for adolescents (126) |
|  |  | 7-day PA recall (60, 64, 89, 91) |
|  |  | 24-hour recall (126) |
|  |  | Child and Adolescent Activity Log (41) |
|  |  | Health Behavior in School-aged Children Survey 2009-2010 (58) |
|  | Device-based | Activity trackers: Fitbit Surge (54), Fitbit Inspire (60), Fitbit Flex (73, 86, 104), Garmin vivofit jr 2 (93), Pedometer (118, 127) |
|  |  | Accelerometers: ActiGraph GT3X (52), ActiGraph GT3X-plus (43, 60, 107, 115), ActiGraph wGT3X-BT (56, 79, 92, 94, 99), ActivInsights GENEactiv (61, 72), Garmin vivofit jr 2 (91) |
| PA Knowledge and awareness | Knowledge | Exercise benefits and barriers scale (50, 59) |
|  |  | Perceived benefits and barriers questionnaires (41) |
|  |  | Knowledge of PA guidelines (17, 20, 89, 119) |
|  |  | Knowledge of other health guidelines (20) |
|  |  | Pre-test and post-test knowledge questionnaires on concepts taught in the video (112) |
|  |  | Knowledge of time spent on PA (76) |
|  |  | Single-item closed response (88) |
|  |  | Knowledge to change lifestyle single-item (67) |
|  | Awareness | Awareness, fun and helpfulness of environmental activities (103) |
|  |  | Brand awareness (118) |
|  |  | Importance of changing lifestyle single-item (67) |
|  | Attitudes | Attitudes to exercising for 5 hours over next 2 weeks (79, 105) |
|  |  | 5-item bipolar adjectives (69, 72) |
|  |  | 2-item exercise attitudes (69, 71) |
|  |  | Towards meeting the guidelines (118) |
| Psychosocial functioning | Self-efficacy for PA | Perceived PA self-efficacy (41, 60, 107, 120) |
|  |  | Modified 18-item self-efficacy scale (123) |
|  |  | PA self-efficacy scale (54, 91, 93) |
|  |  | 5-item subscale of the Health Behavior Questionnaire (73) |
|  |  | Self-efficacy for healthy eating and PA (59, 61) |
|  |  | Adapted exercise self-efficacy scale (77) |
|  |  | Confidence to change lifestyle single-item (67) |
|  | PA motivation | Behavioral Regulations in Exercise Questionnaire (56, 107, 115) |
|  |  | Motivational regulations for school sport (52) |
|  |  | Adapted health care SDT packet perceived competence scale (104) |
|  |  | Exercise motivations inventory (4-items) (64) |
|  | Social support for PA | Researcher developed social network questionnaire (38) |
|  |  | Perceptions of social support for PA (4-items) (40) |
|  |  | School PA support score (40) |
|  |  | Interpersonal influences (41) |
|  |  | Social support scale (104) |
|  |  | Social support for exercise scale (60) |
|  |  | 8-item social support scale (107) |
|  |  | Social beacon network (104) |
|  |  | 23-item Social support survey (126) |
|  | Subjective norms | 2-item Subjective norm (69, 106) |
|  |  | Single-item normative beliefs (71) |
|  |  | 3-items subjective norms (72) |
|  |  | Adapted TPB constructs social norms and perceived physical activity (104) |
|  | PA intentions | 3-item behavioral intentions (69, 72, 120) |
|  |  | 4-item behavioral intentions (52) |
|  |  | 2-item PA intentions (71, 79, 106) |
|  |  | For each movement behavior (119) |
|  | PA planning | 5-item action planning, 5-items coping planning (106) |
|  | Perceived behavioral control | 3-item perceived behavioral control (69, 72, 79, 106) |
|  |  | 4-item perceived behavioral control (71) |
| Intervention engagement & exposure rates | Engagement rates | Attendance logs and dose delivered forms (dose & reach) (39) |
|  |  | Number, type and participation of girls in school-based PA programs (39) |
|  |  | Frequency of use (52) |
|  |  | Facebook engagement data (likes, comments, posts) (86) |
|  |  | % of days wearing & syncing device (86) |
|  |  | Google analytics to determine website viewers (88) |
|  |  | Weekly login rate (59) |
|  |  | % repeat website visits (59) |
|  |  | Frequency of weeks prescribed website login rate was achieved (59) |
|  |  | Technology engagement with the website, Fitbit, texting interactions and Instagram (60) |
|  |  | Intervention exposure rate questionnaire (40, 49) |
|  | Exposure rates | Exposure to promotional materials questionnaire (39) |
|  |  | Exposure to PA outside of class (40) |
|  |  | Exposure to positive messages about PA (40) |
|  |  | Tracking log to document timing and number of each element introduced (48) |
|  |  | Frequency and duration of use of the materials (120) |

*Supplementary file 4- Summary of the RCT studies included in this review in relation to the effectiveness of the methods used to communicate physical activity messages*

| **First author** | **Title** | **Year** | **Purpose** | **Study Design** | **Outcomes measured** | **Summary of findings in relation to effectiveness** | | | | |
| --- | --- | --- | --- | --- | --- | --- | --- | --- | --- | --- |
|  |  |  |  |  |  | **Physical Activity (PA)** | **Psychosocial** | **Intervention exposure** | **Message recommendations** | **Knowledge, awareness, attitudes** |
| Lau | The intervention effect of SMS delivery on chinese adolescent's PA | 2019 | To examine the effects of sms frequency and timing on the efficacy of a SMS-based intervention for hong kong chinese adolescents PA | cRCT | Acceptibility  Exposure  Psychosocial  Self-report PA  stage of change | no significant intervention effects were found among the five groups in PA behavior, stage of change and exercise benefits and barriers among Hong Kong Chinese adolescents. When the SMS frequency and timing issues are treated separately, it is reasonable to assume that the SMS frequency has a positive relation with adolescents’ PA. This finding may offer initial support that five weekly, tailored SMS had a greater impact on self-reported PA than three weekly SMS and the control group. Autonomy of SMS timing could be part of the tailored SMS in which it could fit well into the modern daily life pattern or pace in different cities. | adolescents in the pre-contemplation stage reported the least social support from family and friends. This finding indicated that more social support and encouragement is needed to advance these physically inactive adolescents to the contemplation, preparation and action stages. | 79% of the participants read 1 SMS/week and about half of them responded. The number of SMS read positively correlated with the change in SMR after controlling for exposure to the Internet PA program. | **N/A** | **N/A** |
| Robbins | Intervention effects of ‘girls on the move’ on increasing PA: a group randomized trial | 2019 | To evaluate the effect of a comprehensive school-based intervention in increasing girls minutes of MVPA among fifth to eight grade girls | Group randomized trial | Device measured PA  BMI  Body fat %  process evaluation  Interpersonal influences  activity related affect (enjoyment)  Benefits  barriers  self-efficacy | The GOTM intervention had no significant effect on MVPA at post-intervention or 9-month follow-up. Although the GOTM intervention was well received, findings support researchers’ conclusions in other well-conducted studies that school-based PA interventions alone may be insufficient for helping adolescents to attain and sustain adequate MVPA on their own after the intervention ends. | **N/A** | **N/A** | **N/A** | **N/A** |
| Lubans | Assessing the sustained impact of a school-based obesity prevention program for adolescent boys: the ATLAS cluster randomized controlled trial | 2016 | The aim of this paper is to report the sustained impact of the ATLAS program on primary and secondary outcomes which were assessed 10-months after program completion (i.e., 18-months post baseline). | cRCT | Acceptability  Adiposity  Device-based PA  Sedentary Behavior  Autonomous motivation  Sugar-sweetened beverage consumption  Muscular fitness  resistance training skill competency  motivation for school sport. | The ATLAS intervention was not successful in minimizing the decline in PA that occurs during adolescence. Compliance with accelerometer protocols was poor, making it difficult to draw any firm conclusions regarding changes in PA. Moderator analyses revealed a lack of improvement among low-active adolescents and those who were overweight or obese at baseline. Such findings suggest ‘whole-of-school’ programs may need to be supplemented with targeted programs for the most vulnerable youth. | While ATLAS was successful in preventing a decline in autonomous motivation (intrinsic and identified), controlled motivation increased among participants in the intervention group, with no effect on amotivation. The increase in controlled motivation observed in the current study was an unintended outcome. The ATLAS boys’ higher participation in, and satisfaction with, the teacher-directed sessions as opposed to the student directed sessions suggest that these adolescent boys were still largely motivated by the presence, or pressure, of their teachers. | **N/A** | **N/A** | **N/A** |
| Tessier | The effects of persuasive communication and planning on intentions to be more physically active and on PA behavior among low-active adolescents | 2015 | to examine, using the theory of planned behavior (TPB) combined with a self-regulatory behavior change approach, whether persuasive communication based on adolescents’ salient beliefs (SBCondition) and planning (PCondition) could promote the intention and PA (PA) behavior of low-active adolescents participating in less than 1 h/day of moderate-to-vigorous PA. | Cluster randomized trial- Proof of principle study | self-reported PA  PA intention  subjective norms for PA  perceived behavioral control  Self-reported planning  action planning  coping planning | Even the PC and the Control Condition (CC), which were more effective than the NSBC and the SBC in increasing intention, did not affect PA behavior. | Planning is an effective low-cost intervention in order to increase their intention to be physically more active. | **N/A** | **N/A** | **N/A** |
| Schneider | The effect of a communications campaign on middle school students nutrition and PA: results of the HEALTHY Study | 2013 | To examine the degree to which the communications campaign, which functioned to integrate the various intervention elements and promote visibility of the intervention as a whole, was implemented across the intervention schools and to examine the relation between exposure to the communications campaign and self-reported behavior change among students. | RCT | self-assessment of PA  feasibilty and acceptability of environmental changes  awareness of the campaign messages | The only behavior change that was reported with greater frequency toward the end of the study as compared to baseline was eating behavior consistent with study goals (r = .28, p < .01). Negative trends were found for exercise (r = −.32, p < .01), less TV (r = −.40, p < .001), water (r = −.62, p < .001), and soda (r = −.59, p < .001). When the significance levels were adjusted using the Bonferroni correction for multiple comparisons, significant associations remained between exposure to the campaign and exercise behavior change for the first, second, and last semester and between exposure and both eating and soda drinking for the first semester. | **N/A** | The greatest effect was in the first semester, after that the novelty of the communications campaign began to decline. Intervention schools with higher rates of communication campaign exposure more students reported changing their health behavior. Thus, communication campaign elements may be a valuable adjunct to school-based health promotion efforts. | 1) targeting and responding to multiple audiences, (2) using a broad array of communication modalities, (3) attention to evolving developmental needs of students, (4) awareness of resources required, (5) flexibility and (6) incorporation of local interests. | We saw some decline in students’ reported awareness of communication elements across the course of this 3-year intervention. |
| Fernandez-Escobar | Effect of health-promoting messages in television food commercials on adolescents’ attitudes and consumption: A randomized intervention study | 2021 | this intervention study evaluated the possible health halo effect of HAVISA messages on adolescents aged 11–14 years, as well as their influence on participants’ immediate food choices and predisposition towards healthy eating habits and PA. | Randomized, parallel-group, controlled intervention study | Exposure  healthy choice attitudes towards advertisements  Desire  perceptions of healthiness  advisable frequency  intention to consume  importance of a healthy diet  desire for PA desire for fruit desire for veg | This research suggests that health-promoting messages have small to no positive immediate effects food behaviors, and that they lack immediate negative, “health halo” effects. | Health-promoting messages have small to no positive immediate effects on health-related attitudes | Only a small percentage of participants correctly recognized the messages shown, which suggests that low prominence is partly to blame for health-promoting messages’ lack of immediate effectiveness. | **N/A** | **N/A** |
| Hill | Can theory-based messages in combination with cognitive prompts promote exercise in classroom settings? | 2007 | The present study evaluated a leaflet designed to encourage increased exercise amongst a youth sample using persuasive text to change attitudes, norms and behavior control. The study also examined the extent to which cognitive changes mediated changes in self-reported exercise. | RCT | Exercise  Attitudes  Intention  behavior control  normative beliefs | leaflet increased reported exercise and this effect was greatest for students who (1) had weaker pre-intervention intentions to increase exercise, (2) reported less control over being able to do so and (3) had a less positive attitude towards doing additional exercise. | **N/A** | **N/A** | **N/A** | **N/A** |
| Aceves-Martins | "Som la Pera", a school-based peer-led social marketing intervention to engage spanish adolescents in a healthy lifestyle: a parallel-cluster RCT | 2022 | to assess the effectiveness of the "Som la Pera" intervnetion in promoting an increase in PA, fruit and veg consumption and reduce screen-time in adolescents. | School-cluster, randomized, controlled, parallel study | Fruit and vegetable intake  Self-reported Moderate to vigorous intensity PA  breakfast consumption  obesity prevalence  engagement of local organizations as stakeholders. | A school-based, peer-led SM intervention designed and implemented by adolescents attending high schools in low-income neighborhoods effectively improved the PA, particularly in males. No effect on fruit or vegetable consumption or screen time per day was observed. | **N/A** | The Facebook page created by the Activity Challenge Creators (ACC’s) received 496 likes. Of the 169 participants in the intervention group, 113 (66.9%) followed the Facebook page, 39 (23.1%) did not follow the page, and 17 (10.1%) did not have a Facebook account or used a different nickname on their social media account. These numbers indicated only followers of the Facebook page, while several interactions among adolescents who did not follow the page were found throughout the study during the Facebook activities, such as using the information posted to the classroom wall or schoolmates' internal chats. In addition, none of the students in the control group followed the “Som la Pera” Facebook page. | **N/A** | **N/A** |
| Marks | A Comparison of Web and Print Media for PA Promotion among Adolescent Girls | 2006 | To compare the effectiveness of a Web-based PA (PA) intervention with identical content delivered in a printed workbook among a sample of adolescent girls. | randomized trial design | Self-reported PA  self-efficacy  intention for PA | Low active girls increased self-reported MVPA and the change was greater in the print media group. | Increased PA self-efficacy and intentions. | **N/A** | This suggests that differences in the mode of delivery may account for the variability in communication and behavior outcomes. A possible explanation for the differential effectiveness of print and Web media is that information is processed differently between the two media. One hypothesis for this difference is that print may be perceived as a didactic media among school-age children who still learn primarily from text books and printed materials, whereas the Web may be perceived as an entertainment media and therefore have less credibility and/or facilitate less attention to the messages. | **N/A** |
| Moitra | Impact of a behaviorally focused nutrition education intervention on attitudes and practices related to eating habits and activity levels in Indian adolescents | 2021 | To evaluate the effectiveness of a behaviorally focused nutrition education intervention based on the Health Belief Model to improve knowledge, attitudes and practices related to eating habits and activity levels in 10-12 year old adolescents | Cluster randomized control trial | Knowledge  Attitude  Diet  self-reported PA | A mean improvement is 9.6% in diet and 9.4% in PA scores from pre to post- intervention. | N/A | N/A | N/A | intervention group reported mean improvement of 39.3% in knowledge of PA and attitudes increased by a mean of 7·3 % |
| Newton | Pedometers and Text Messaging to Increase PA | 2009 | To assess whether pedometers and text messaging increase PA in adolescents with type 1 diabetes | Randomized control trial | PA–- steps self-reported  Adherance–- through charts with step count collected at follow up  blood pressure  BMI Z score  Quality of Life. | Using pedometers and text messaging as motivational tools in adolescents with T1DM did not increase PA. Mean daily step counts reduced significantly in the control group and only a little in the intervention group after the intervention. Self reported MVPA increased by 38.5 min/week in control group and 48.4 min/week in the intervention group. | **N/A** | **N/A** | **N/A** | **N/A** |
| Lubans | Preventing Obesity Among Adolescent Girls: One-Year Outcomes of the Nutrition and Enjoyable Activity for Teen Girls (NEAT Girls) Cluster Randomized Controlled Trial | 2012 | The aim of the current study is to evaluate the effects of the Nutrition and Enjoyable Activity for Teen Girls (NEAT Girls) program | RCT | BMI (wt and ht)  body fat %  muscular endurance  core-abdominal isometric muscular endurance  Device measured PA levels  Sedentary Behavior questionnaire  physical self-description | the NEAT Girls intervention resulted in small improvements in body composition and large reductions in self-reported screen time but did not increase their PA. | **N/A** | **N/A** | Although NEAT Girls was not targeted toward a specific cultural group, the importance of addressing cultural uniqueness is relevant to our study and we employed a number of strategies to ensure that the intervention was tailored and relevant to the participants. For example, the intervention logo and materials were branded and tailored to appeal to adolescent girls. A variety of novel strategies were used to engage girls in the interactive seminars (eg, game show format) and participants were encouraged to bring their own music to be played on a portable digital music player in the enhanced school sports sessions. | **N/A** |
| Patrick | Outcomes of a 12-Month Technology-Based Intervention to Promote Weight Loss in Adolescents at Risk for Type 2 Diabetes | 2013 | to evaluate the effectiveness of an intervention targeting this population that was offered to participants recruited through clinical sites but primarily delivered through combinations of three modalities: the web, group sessions for adolescents and parents, and short message service (SMS). | RCT | anthropometric  self-reported behavior measures  self-reported psychological measures  behavior change strategies | Adolescents in the Web arm decreased their sedentary behavior from 4.9 to 2.8 average hours per day compared with the Usual care group, which only decreased from 5.4 to 5.3 average hours per day of sedentary behavior at 12 months (p = .006). | **N/A** | **N/A** | **N/A** | **N/A** |
| Bandeira | Implementation of a school-based PA intervention for Brazilian adolescents: a mixed-methods evaluation | 2022 | this study aimed to evaluate the implementation of a school-based PA intervention in Brazil, considering both qualitative and quantitative data from the different actors (students, teachers and parents) inherent to the implementation of the program. | Mixed methods cRCT implementation evaluation | Teacher training  educational strategies  environmental improvements | According to 79%, 42% and 22% of the teachers, students and parents, respectively, the messages delivered through the pamphlets were able to positively alter the lifestyle of the students. | **N/A** | Most of the teachers (68%) reported holding classroom health discussions, but 52% of the students did not notice these discussions | **N/A** | **N/A** |
| Tessier | The effects of a cluster-randomized control trial manipulating exercise goal content and planning on PA among low-active adolescents | 2022 | In sum, the first study was a pilot study that aimed to develop and test the two goal-framing messages, and the second study was the main trial including a control condition, a planning intervention, an objective measure ofPA behavior, and a mediation analysis. | cRCT | **Study 1**: Self reported PA; Intention: Perceived behavioral control; attitude; exercise goal content. **Study 2:** Same as study 1 with the addition of a direct PA measure | compared to low-active adolescents in Control Condition (CC) , those in Extrinsic Message Condition + Planning (EMC+P) and Intrinsic Message Condition + Planning (IMC+P) did not practice more MVPA, but carried out more LPA. Path analyses revealed three main findings: 1. “CC vs. EMC + P-IMC + P” was significantly related to LPA. | compared to IMC + P and CC, EMC + P produced an increase of perceived behavioral control. The framing effect on intention decreased rapidly. While in the two experimental conditions follow-up scores of intentions – taken 2 weeks after the intervention – were significantly higher than the pre-test scores, they significantly decreased from post-test to follow-up. | **N/A** | **N/A** | **N/A** |
| Walters | Exploring attention to the Canadian 24-Hour Movement Guidelines for Children and Youth using eye-tracking: A randomized control trial | 2022 | (1) to determine if branding the Guidelines affects youths’ attention to the Guidelines, brand perceptions, and Guideline cognitions (awareness, recall, attitudes, and intentions). (2) to explore the relationship between attention and brand perceptions and Guideline cognitions. (3) to explore the brand features that draw youths’ attention. | Cross-sectional between participants randomized intervention design | Brand perceptions  guideline cognitions- recall  attitudes  intentions  attention- fixation count  dwell time  run count | N/A | the branded Guidelines neither drew greater overall attention nor led to more positive brand perceptions or Guideline cognitions compared to the unbranded Guidelines. | Duration and frequency of guideline exposure is a key methodological limitation. Although youth were free to view the Guidelines for as long as desired, they chose to view the materials for approximately 80 s only. Moreover, the Guidelines were presented in a single exposure. | The materials were not tailored to the population. We used Guideline materials drawn from standard practice. These materials are written for a broad audience with a reading level far exceeding reading levels of 10–14-year-old youth. | **N/A** |
| Willinger | Digital Health Nudging to increase PA in pediatric patients with congenital heart disease: A randomized controlled trial | 2023 | This randomized controlled trial (RCT) aimed to increase PA, health-related quality of life (HrQoL), as well as activity-related self-efficacy (ArSE) in adolescents with CHD by a 12-weeks Dig ital Health Nudging intervention. | RCT | PA  HRQoL  Activity related Self efficacy | This randomized control trial shows that 12-weeks of Digital Health Nudging did not increase PA and ArSE. | improved feelings of emotional well-being in adolescents with CHD.  No increase in activity related self-efficacy. | **N/A** | Need to individualise nudges to age, baseline activity level | **N/A** |
| **PA- Physical activity** | | | | | | | | | | |

*Supplementary File 5- Additional quotes from stakeholder consultation*

| **Topic area** | **Quote** |
| --- | --- |
| Findings that stood out to them | *“That's something that I expected, and it is confirmed by your findings, that it can have an impact on their knowledge awareness. But it could be that you need more to really change the behavior, which is of course not a bad thing because this is only a part and it's the start. It's already good that you can increase knowledge or awareness” ~* Researcher. |
|  | *“Supposed for me as school principal is definitely an awareness there but am I doing the right thing, I think it’s the right thing, but is it the right thing and are some of the messages I'm sending out my role? Would I be indirectly doing damage? And now that I come away and think about it there’s definitely awareness there, you know, umm, this message we are trying to create is it on the right thing or is it ultimately doing damage to the kids perception and knowledge and behaviors and there's a really impact” ~* Secondary school management. |
| Differences between findings and practice/policy | *“The lads will be quick to say yeah, I’ll give it a go… maybe there’s a fear of failure with girls.. imagine tripping up and falling down over a football or rugby ball but in front of their peers particularly for the girls, it is hard” ~* Secondary school management. |
